# Horticulture in a molecular age

**DOI:** 10.1186/s43897-021-00007-8

**Published:** 2021-06-16

**Authors:** Su-Sheng Gan, Hong-Wei Xue

**Affiliations:** 1 Plant Biology Section, School of Integrative Plant Science, Cornell University, Ithaca, NY 14853 USA; 2 Joint Centre for Single Cell Biology, School of Agriculture and Biology, Shanghai Jiao Tong University, Shanghai, 200240 China

It is our great honor and pride to announce and celebrate the launch of **Molecular Horticulture** (MHort). On behalf of the distinguished Advisory Board and Editorial Board of the journal, we would like to warmly welcome you to visit the MHort webpage and read the first batch (and more to come) of articles of high quality and impact.

**Why is this new journal needed?** The word *horticulture* derives from two Latin terms, *hortus* (meaning “yard” or “garden”) and *cultura* (meaning “cultivation”), and *Horticulture* has been conventionally a practice- or application-oriented discipline. The utilization of molecular biology and related techniques in horticulture research has led to the new frontier or branch of horticulture – *Molecular Horticulture* – and much related research has been performed, resulting in the significant increase in research papers. For example, the improvement of sequencing technology, especially the single molecular real time sequencing approach (SMRT)-based third generation of sequencing (such as the Nanopore sequencing), with drastically reduced costs, has made it possible to have genomes of more and more horticultural plants sequenced. In 2007, only one horticultural plant’s genome was sequenced; in contrast, 42 horticultural plants’ genomes were sequenced in 2017 alone. Some other examples include the cutting-edge mass spectrometry (MS) and single cell techniques that have also significantly transformed and advanced research at the molecular level in horticultural plants.

The increasing use of the molecular genetic tools and related techniques has rapidly increased the number of related publications, for instance, from ~ 3000 in 2002 to ~ 12,000 in 2012 resulting from the application of genomic technologies, and the increase in the number is believed to be accelerating. Although several new horticulture journals have recently been started, the current journals cannot meet the needs of the molecular horticulture research output. MHort has now launched to fulfill the high demand (albeit partially). On the other hand, there are increasingly high-quality researches on horticultural plants that deserve publication in prestigious journals, but there are only few such high-impact horticulture journals. MHort has now launched to meet the needs. It is our hope that MHort will become the premier horticulture journal with the most profound impact on horticulture field.

**What does the journal publish?** As you will find from the Scope, the topics covered by MHort are very broad, from (eco) physiology, biochemistry, and cell biology, to synthetic biology, (epi) genetics, and multi-omics of horticultural plants. Horticultural plants are also loosely or inclusively defined and include medicinal plants. In addition, the journal will also publish research articles involving model plants that reveal mechanisms/principles readily applicable to horticultural plants. The journal also welcomes resources and methodology articles that significantly facilitate or revolutionize horticultural research.

**Who runs the journal?** MHort is run by scientists. Specifically, the journal is guided by the distinguished Advisory Board consisting of preeminent, world-class academics, and is edited by the distinguished international Editorial Board comprised of outstanding scholars. It is highly expected that the board will be expanded and dynamic. Our board members are very excited about and ready to help you in publishing your excellent manuscripts in MHort. Most importantly, the journal is also run by you, our very authors and dear readers! We sincerely hope that you will help the journal to excel by submitting your excellent research and review articles, by serving as reviewers and by becoming a frequent reader of MHort.

**Why should you consider publishing in MHort?** In addition to reasons mentioned above, MHort is **Open Access** (**OA**) yet **free of charge**. Briefly, in partnership with Springer Nature, MHort is made fully **Open Access** so your articles can be readily accessed by as many readers as possible worldwide. In addition, **no fee** will be levied on the authors of MHort for the first three years for sure.

MHort is ready to take off! And we are very much looking forward to having you on board as an author, a reviewer, and/or a reader!

## Authors’ information

Su-Sheng Gan and Hong-Wei Xue are Editor in Chief of Molecular Horticulture.

